# A Network View on Parkinson's Disease

**DOI:** 10.5936/csbj.201304004

**Published:** 2013-07-14

**Authors:** Sreedevi Chandrasekaran, Danail Bonchev

**Affiliations:** a Center for the Study of Biological Complexity, Virginia Commonwealth University, United States

## Abstract

Network-based systems biology tools including Pathway Studio 9.0 were used to identify Parkinson's disease (PD) critical molecular players, drug targets, and underlying biological processes. Utilizing several microarray gene expression datasets, biomolecular networks such as direct interaction, shortest path, and microRNA regulatory networks were constructed and analyzed for the disease conditions. Network topology analysis of node connectivity and centrality revealed in combination with the guilt-by-association rule 17 novel genes of PD-potential interest. Seven new microRNAs (miR-132, miR-133a1, miR-181-1, miR-182, miR-218-1, miR-29a, and miR-330) related to Parkinson's disease were identified, along with more microRNA targeted genes of interest like *RIMS3, SEMA6D* and *SYNJ1*. David and IPA enrichment analysis of KEGG and canonical pathways provided valuable mechanistic information emphasizing among others the role of chemokine signaling, adherence junction, and regulation of actin cytoskeleton pathways. Several routes for possible disease initiation and neuro protection mechanisms triggered via the extra-cellular ligands such as *CX3CL1*, *SEMA6D* and *IL12B* were thus uncovered, and a dual regulatory system of integrated transcription factors and microRNAs mechanisms was detected.

## 1. Introduction

James Parkinson has been the first to observe this disease in adults in the year 1817. In his essay entitled “An Essay of the Shaking Palsy” he described this disease as initiated with slow, progressive involuntary tremors, followed by difficulty in walking, swallowing and speech [1]. Apart from motor symptoms, Parkinson's disease patients experience significant non-motor symptoms including mood and cognition decline, sleep disturbances, and other autonomic dysfunctions [2]. With the help of modern-day molecular and cellular research advancement, progressive degeneration of the dopaminergic (DA) neurons of the Substantia nigra (SN) brain region were found in Parkinson's disease brains [3], in addition to the accumulation of misfolded protein aggregates. Both environmental factors and genetic mutations were suspected to cause PD [4, 5]. One of the distinctive features of Parkinson's disease is severe damage to the nigrostriatal dopaminergic system. Neurotoxic agents such as manganese and 1-methyl-4-phenyl-1,2,3,6-tetrahydropyridine (MPTP) were suspected for this type of neuronal damage. MPTP induced Parkinson's disease animal models were extensively used to study the neurodegeneration process as well as to identify potential therapeutic drug targets [6]. Soluble fractalkine (*CX3CL1*, chemokine ligand 1) isoform was shown to reduce impairment of motor coordination, decrease dopaminergic neuron loss, and ameliorate microglial (macrophages of brain) activation and proinflammatory cytokine release resulting from MPTP exposure [7].

**Figure 1:**
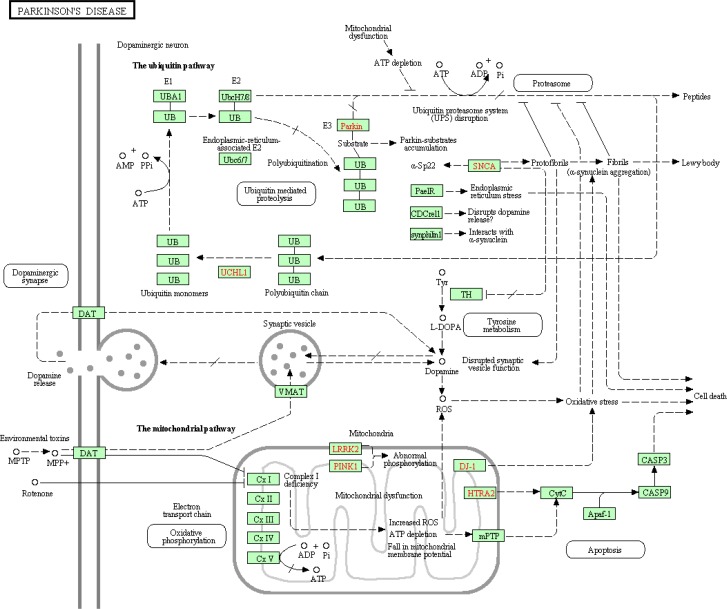
**Biological processes and genes implicated in the Parkinson's disease**. Courtesy: Parkinson's disease pathway from KEGG database, retrieved on April 3, 2013.

Long time belief was that Parkinson's disease etiology is sporadic (not genetically inherited) in nature. However, a small percentage of the PD patients were now known to inherit gene mutations. Genes including *ATP13A2*, *DJ-1*, *GIGYF2*, *HTRA2*, *LRRK2*, *PARK2* (parkin), *PINK1*, *SNCA* and *UCHL1* were associated with either autosomal dominant or recessive form of Parkinson's disease [5]. From the listed genes *SNCA* (α-synuclein or α-syn) is critical to the pathogenesis in the early-onset of the rare familial form of PD. Insoluble form of α-syn fibrils were discovered in the protein aggregates called Lewy bodies (LBs), the hallmark pathological characteristics of Parkinson's disease. The aggregation and accumulation of abnormal α-syn in dopaminergic neurons have been postulated to be responsible for the neurodegeneration that ultimately leads to cell death [8, 9]. Synucleins were also found in the amyloid-plaques in Alzheimer's disease brains. In general, alpha-synuclein is highly expressed in brain at presynaptic terminals, particularly in the neocortex, hippocampus, striatum, thalamus, and cerebellum components. They function as molecular chaperones and interact with many proteins thus modifying their cellular activity. Due to its versatile interacting behavior, mutant alpha-synuclein has been implicated in the deregulation of many biological processes including oxidation, neuroinflammation, mitochondrial function, ubiquitination etc. [3, 10–12]. Figure 1 depicts the various genes already implicated in Parkinson's disease along with different deregulated biological processes caused by the several abnormal protein activities.

To date, many genetic modifiers of PD and their role in PD pathogenesis have been described [13–17]. Some of these genes relate to neuronal growth and neuroprotective mechanisms in Parkinson's disease. FGFs (fibroblast growth factors) have potent neurotrophic properties for dopaminergic neurons [18]. They promote DA neuron's development and neurite outgrowth, rescue damaged DA neurons after different toxic insults, and prevent apoptosis. Overexpression of *L1CAM* (L1 cell adhesion molecule) enhances the survival of imperiled endogenous dopaminergic neurons in the Substantia nigra [19]. *RAB3A* (member of RAS oncogene family) has been shown to suppress α-syn toxicity in neuronal models of PD [20]. Fractalkines produced by neurons suppress the activation of microglia and play a neuroprotective role in 6-OHDA-induced (synthetic neurotoxic compound) dopaminergic lesions [21]. In general, metallothioneins (cysteine-rich, heavy metal-binding protein molecules) have been considered ‘defensive proteins’ with a role in neuroprotection. Metallothioneins 1 and 2 (*MT1F*, *MT2A*) have been shown to scavenge reactive oxygen species and free radicals in central nervous system [22].

Other genes have been implicated in PD pathogenesis. Neuroinflammation is suspected to play a major role in Parkinson's disease progression. MAPK signaling pathways contribute to neuroinflammatory responses and neuronal death triggered by synuclein-alpha aggregates or functional deficiencies in parkin or *DJ-1* genes in the pathogenesis of PD [23]. *RNF11* (ring finger protein 11) was suggested to play major role in the Parkinson's disease pathology since it was found highly enriched in SN dopamergic neurons as well as its co-localization within Lewy bodies (abnormal aggregates of protein) in PD brains [24]. Earlier study by Galvin et al., (1999) had shown that β- and γ-synuclein are associated with hippocampal axon pathology in Parkinson's disease and dementia with Lewy bodies [25]. Recent genome-wide studies have found that mutations in at least 13 *PARK* loci and related genes increase both early- and late-onset PD susceptibility [15, 26, 27].

Genome-wide approaches were also used to identify microRNAs-target mRNA interactions in PD domain. MicroRNAs (miRNAs) are a class of small RNAs (∼22 nucleotides) that act as post-transcriptional regulators of gene expression by binding to the complementary sequences in target mRNAs. In recent years, miRNAs have emerged as potential drug targets in a variety of diseases including infections, metabolism and inflammation etc. [28]. A recent genome-wide miRNA profiling study for Parkinson's disease has reported several miRNAs to be differentially expressed in PD blood samples. The hundreds predicted genes targeted by these miRNAs belong to various biological pathways including synaptic long-term potentiation, semaphorin signaling in neurons and protein ubiquitination pathway, etc., many of which were previously found deregulated in Parkinson's disease mechanism [29].

Even though there were some new treatment options available to PD patients, oral administration of levodopa (precursor of dopamine) has been the gold standard medication for Parkinson's disease. But prolonged use of levodopa increases the risk of developing levodopa-induced dyskinesias (involuntary movement) [30, 31]. Recently, deep brain stimulation (DBS) has been offered as a secondary treatment option in Parkinson's disease where the benefits of medication have failed/diminished. DBS therapy has been shown to increase the neuron firing rate, blood flow and to promote neurotransmitter release as well as to stimulate neurogenesis. Although deep brain stimulation improves the motor symptoms of Parkinson's disease, it is a serious surgical intervention with major side effects of infection and intracranial hemorrhage including the risk of death [32].

In our study we construct a variety of biomolecular networks proceeding from several gene expression datasets covering different areas of brain affected by Parkinson's disease. Two such sets were reported by Moran et al. in 2006, who provided a whole genome analysis of the Substantia nigra (SN), found considerable difference in the gene expressions compared to control, reported several new genes that map to *PARK* loci, and identified 570 “priority genes” after using the Benjamini-Hochberg FDR correction [33]. Two years later, the same group published a network-based analysis based on Pathway Studio's ResNet database version 5.0. Several *direct* interaction networks have been constructed for the interactions between priority and known-PD genes. Cancer, diabetes and inflammation disease conditions have been associated with the top up-regulated priority genes. Another set was published by Zhang, et al. in 2005 [34], highlighting some of the deregulated genes responsible for either disease aggravation (*MKNK2*) or neuroprotection (*HSBP1*, *SMA5*, and *FGF13*). Deregulation was noticed in various genes belonging to metallothionein group and the heat shock protein group. These patterns of multiple molecular process deregulations have been found across different brain regions studied. Another expression pattern discovered supports the hypothesis for ubiquitin/proteasome system (UPS) dysfunction in Parkinson's disease. A decrease in Complex I activity has also being found to reinforce the suspected mitochondrial deregulation in PD.

With current advancement of different “omics” technologies along with effective in-silico testing options, finding successful molecular therapeutic targets for Parkinson's disease seems much closer than before. Along this avenue, the current paper presents a comprehensive network-based analysis of Parkinson's disease (PD) related microarray datasets. Helped by the latest accumulated knowledge of gene/protein interactions and sophisticated software for network analysis we were able to expand upon the previous analyses of this disease paradigm, underlying cellular mechanisms and critical molecular players, as well as to identify novel drug targets. This research work on Parkinson's disease is part of a broader network-based data analysis of three neurodegenerative disorders (NDDs) including Alzheimer's (AD) and Huntington's disease (HD) with the final goal the identification of unified underlying molecular mechanisms of these three devastating NDDs. Manuscripts outlining our research findings of AD and HD, including the unified molecular mechanisms of NDDs, are in preparation and will be submitted for publication subsequently.

## 2. Methods and Data

**Figure 2:**
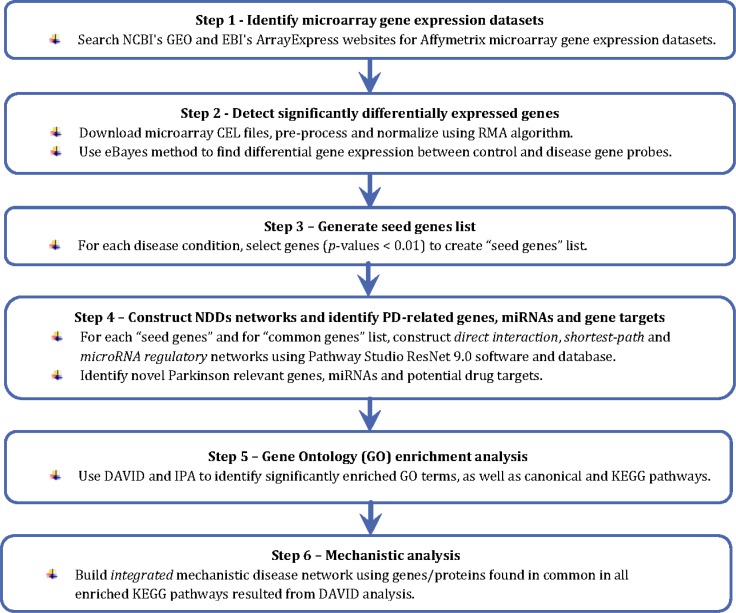
The study workflow.

The work flow followed in this study is illustrated in Figure 2.

### Step 1: Microarray gene expression data

DNA microarray is a powerful technology that provides a high throughput and detailed view of the entire genome and transcriptome of an organism by measuring the relative mRNA abundance intensity. Due to their ready availability, high volume capacity and parallel testing, microarrays have dramatically accelerated many types of molecular biology investigation. The known limitation of using microarrays is that mRNA level does not necessarily correlates with its functional protein level in the cell. Also, post-translational modifications essential for determining protein function are not present on DNA microarray. However, these limitations could be partially overcome by careful handling of arrays, probe selections and repeat experiments. Moreover, microarray assays are inexpensive and less-time consuming when compared with proteomics experiments. Better results in understanding the underlying biological mechanisms are yielded by integrating gene expression along with proteomics data. Such high quality proteomic data are expected to be provided by the Human Brain Proteome Atlas, a project launched by 10th HUPO World Congress in 2011 [35].

Microarray gene expression of post-mortem brain tissue samples from diseased and control conditions were used. The three Affymetrix GeneChips sets used were GSE8397 U133A and U133B (http://www.ncbi.nlm.nih.gov/geo/query/acc.cgi?acc=GSE8397) and GSE20295 U133A (http://www.ncbi.nlm.nih.gov/geo/query/acc.cgi?acc=GSE20295) arrays. This specific selection was influenced by our extended research plan to search for the unified underlined mechanism of neurodegenerative diseases. Only Affymetrix post-mortem datasets were found to cover the three most characteristic neurodegenerative diseases - Parkinson's, Alzheimer's and Huntington ones. The samples were initially selected after careful review of the cases neuropsychological and/or neuropathological data, and matched by age and sex. The control subjects were with no known neurodegenerative disease history. The GSE8397 arrays included 15 cases and 8 controls each with male to female ratios 9:6 and 6:2, respectively. The mean age of cases reported was 80±5.7 whereas that of controls 70.6±12.5. The brain tissues/regions involved were Superior Frontal Gyrus (SFG), Medial Substantia Nigra (MSN) and Lateral Substantia Nigra (LSN). The GSE20295 array has equal number of 15 cases and controls. The male to female ratios for the two groups were 9:6 and 10:5, while the mean age was 76.7±6.2 and 71.2±11.1, respectively. Broadman Area 9 (BA9), Putamen (PT) and Substantia Nigra (SN) were the brain tissue/regions involved.

### Step 2: Detection of significantly differentially expressed genes

The microarray data analysis was focused on genes differentially expressed across different tissues. For consistency between the selected datasets, the latter were subjected to the same techniques for pre-processing, normalizing and post-normalizing. Bioconductor software for analysis and comprehension of genomic data based on R programming language [36] (http://www.bioconductor.org/) was implemented in written in-house R. The raw microarray CEL files were downloaded from the GEO/ArrayExpress databases, and the microarray chip quality was assessed using *arrayQualityMetrics* [37]. More specifically, GeneChip reproducibility was assessed, signal-to-noise ratio was determined and no extreme outliers were detected. Relevant quality assessment figures/plots were obtained

All microarray expression datasets were normalized to correct for systematic differences due to sample preparation, batch processing, etc. between genes or arrays. A multi-array average (RMA) expression measure was used [38], which consists of three steps: background correction, quantile normalization (each performed at the individual probe level), and robust linear model fit using median polish (log-transformed intensities at the probesets level). The standard RMA approach was utilized to enable more direct comparisons with other similar research results.

The differential gene expression changes were statistically evaluated by the empirical Bayes (eBayes) method [39] from the *limma* Bioconductor package. Probe-sets with *p*-values < 0.05 were considered to be significantly differentially expressed genes (SDEGs). R source code for statistical analysis of such microarray gene expression dataset, including graphical output of the differentially expressed gene, can be obtained from the authors by request.

### Step 3: Generation of “Seed genes” Set

**Figure 3:**
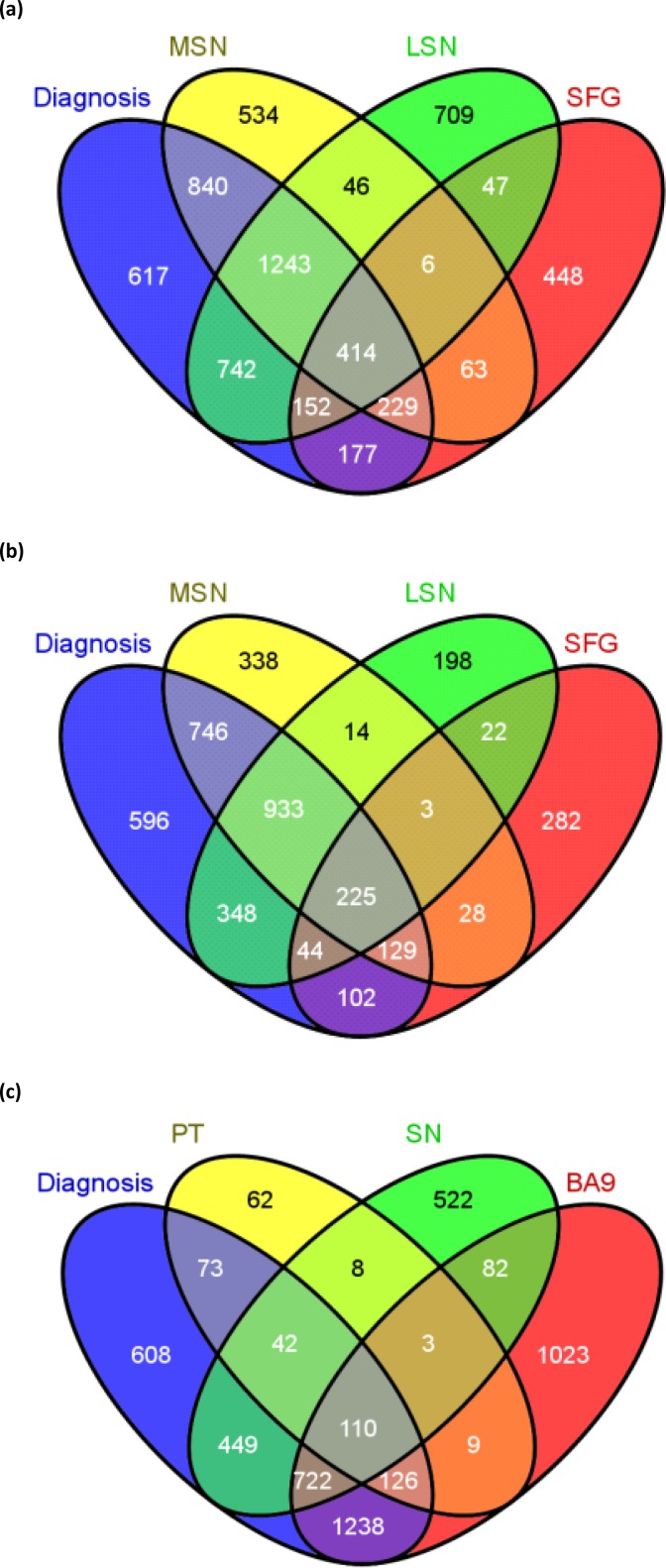
**Four-set Venn diagram of the overlap of significantly differentially expressed genes (SDEGs)** in (a) GSE8397 HG-U133A (b) GSE8397 HG-U133B and (c) GSE20295 HG-133A gene expression datasets.

The microarray datasets were subjected to the same statistical procedures given above and significantly differentially expressed genes lists called “seed genes” were generated for each dataset. The lists generated from the GSE8397 dataset were denoted as SFG, MSN and LSN, for the three types of brain tissue samples: superior frontal gyrus, medial and lateral Substantia nigra, respectively. In addition, differential gene expression changes found between control and PD cases irrespective of tissue types were denoted as “*Diagnosis*”. An overlap of 414 seed genes was found between the four sets of significantly differentially expressed genes (SDEGs), as shown in Figure 3a. In a similar way, an overlap of 225 seed genes was found in the GSE8397 HG-U133B microarray gene expression dataset (Figure 3b). Altogether, 631 seed genes were found after removing duplicates in GSE8397 U133A and U133B datasets.

Correspondingly, using GSE20295 HG-U133A (Figure 3c) microarray dataset another four sets of seed genes, namely *diagnosis, BA9, PT and SN* (for tissue samples used), were generated and an overlap of 110 genes were considered SDEGs (*p*-values < 0.05). Finally, combining the three Parkinson's microarray datasets (GSE8397 (HG-U133A and B) and 20295) we found a total of 719 (*p*-values < 0.05) genes to be significantly differentially expressed.

The p-values shown above were obtained with paired p-test without correction for multiple correlations. Due to the specificity of the post-mortem expression datasets no statistically significant expressed genes were found after Bonferoni correction, while with the less stringent Benjamini-Hochberg correction the number of SDEGs was not large enough to allow for meaningful analysis. However, to compensate partially for not taking into account the probes correlation, we selected to consider a more stringent p-value cut-off of 0.01 for the paired p-test value. With the new cut-off the total number of “seed genes” for Parkinson's disease was reduced from 719 to 267 (see Supplementary Table S1 for details).

### Step 4: Construction of various types of neurodegenerative disorder networks. Identification of novel PD-genes and drug targets

For constructing and analyzing networks relevant to neurodegenerative diseases we selected Pathway Studio 9.0 software package [40], (http://www.elsevier.com/online-tools/pathway-studio). It offers options to construct various kinds of networks such as direct interaction, shortest-path, common targets and regulators of pairs or multiple genes, and others. The molecular interaction data used in the study were supplied by the ResNet 9.0 database (released October 15, 2011), provided jointly with the software. It covers human, mouse and rat proteins. The database is compiled by using MedScan technology from over 20 million NCBI's PubMed abstracts and over 880,000 full-text articles as of May 27, 2011. Currently the database covers 125342 entities, such as cell process, complex, disease, functional class, treatment and small molecules including over 110000 genes/proteins. It offers over a million interactions like binding, chemical reaction, direct regulation, expression, miRNA regulation, molecular synthesis, molecular transport, promoter binding, protein modification and regulations, as well as information about almost 5600 custom built cell-processes, metabolic and signaling pathways.

In this study, we constructed *direct interaction* (*DI*) and *shortest-path* (*SP*) networks to analyze interactions between the SDEGs and with connecting genes/proteins that could be of interest in neurodegeneration process. Direct regulatory interactions of five different types were used only, including among others promoter binding, protein modification and miRNA regulation

By applying the *shortest-path* (SP) network strategy with the list of SDEGs, we were able to identify connecting genes/proteins that might contribute to the neurodegenerative process but have not been related so far to it. This approach is based on the inference that genes/proteins with well-defined biological functions when interacting with other genes/proteins known of importance for given disease like Parkinson's have a higher probability to share that function, as compared to those selected at random (guilt-by-association). One limitation of the shortest-path network approach is that sometimes it could bring in a large amount of intermediary nodes in order to have a unified network. Such a huge network is not only impractical for further analysis, but it also diminishes the importance of the seed genes in the selected scenario. Thus, care was taken to reduce the number of connecting nodes in the shortest-path network producing *compact shortest-path* networks. The last task was accomplished by setting up a cut-off rule, to include only seed genes with a large number (≥ 25) of neighbors in the Pathway Studio ResNet 9.0 database, thus focusing on genes having a better chance to be connected to known Parkinson's disease genes. The 267 seed genes were thus reduced to 105 genes and the ratio of connecting to seed genes/proteins ranged from 1.5:1 to 2:1 for all the datasets. The construction of the compacted SP network was finalized by adding few generic genes without which some of the genes of interest would still remain unconnected.

Special attention in the network analysis was paid to identifying the key players - nodes with high network topology scores of node degree (local connectivity), closeness centrality (network monitoring) and betweeness centrality (traffic-influential) scores [41]. The calculation of these topological descriptors was executed with the Pajek software package [42]. Nodes with such favorable topological characteristics, along with biological/ molecular functions relevant for the neurodegenerative process, have been considered in two categories “already known PD-genes” and “genes of interest for PD”. The distinction was made by using sources like Online Mendelian Inheritance in Man (OMIM) database (http://omim.org/), NCBI's PubMed database (http://www.ncbi.nlm.nih.gov/pubmed.com), MalaCards database (http://malacards.org/), and Google search for the latest publications (http://www.google.com). Each of these two categories were further divided in two subcategories, those found among the significantly differentially expression genes (SDEGs) and such emerging from the connecting proteins in shortest-path and common regulator networks.

Differential gene expression was analyzed through complex regulatory networks that are controlled by two types of regulators: transcription factors (TFs) and microRNAs (miRNAs). In order to identify the microRNAs that target our seed genes we constructed *shortest-path* network with only miRNA regulation type of interactions using Pathway Studio's ResNet 9.0 database.Then, in order to construct a miRNA regulatory network we used the *direct interaction* network option in Pathway Studio utilizing the seed genes and the corresponding miRNAs identified in the earlier step. We identified many microRNA regulations of our seed genes which will be discussed in detail in the following sections. The microRNA regulatory network also revealed an integrated regulation in neurodegeneration process by both transcription factors and microRNAs. However, the miRNA regulatory analysis should be offered with some caution, because currently a high percentage of miRNA-mRNA interactions in Pathway Studio ResNet 9.0 database are based on predictions but not on experimental validation.

### Step 5: Gene Ontology (GO) analysis. Identification of enriched pathways

Gene ontology (GO), an expert-curated database, assigns a list of genes into various biologically meaningful categories such as biological process, molecular function, and cellular component. *p*-values are used to rank the significantly modulated genes into GO categories. We used the Database for Annotation, Visualization and Integrated Discovery (DAVID) [43–45], which provides biological functional interpretation of large lists of genes derived from genomic studies such as microarray, proteomics experiments, etc. Core analysis in Ingenuity's IPA (Ingenuity Systems, www.ingenuity.com) and Pathway Enrichment Analysis in Pathway Studio were then applied to identify enriched canonical pathways in Parkinson's disease, and the genes from the lists of SDEGs and network generated lists that take part in the enriched pathways.

### Step 6: Mechanistic Analysis

The results from DAVID analysis were examined in an attempt to characterize the *integrated* molecular mechanisms involved in neurodegeneration process. The output includes those GO categories and KEGG pathways that are enriched in a given list of genes. Kyoto Encyclopedia of Genes and Genomes (KEGG) is a basic database resource for understanding high-level functions of biological systems from molecular-level information, especially large-scale molecular datasets generated by genome sequencing and other high-throughput experimental technologies (http://www.genome.jp/kegg/) [46]. The KEGG pathways that were significantly enriched (p-value ≤ 0.05 after Benjamini-Hochberg FDR adjustments) and previously known in neurodegenerative disorders under study were identified and further investigated. Google and NCBI's PubMed databases were used to search for such previously known biological pathways in neurodegenerative disorders. After that, all the genes from the enriched KEGG pathways were combined into a list of “mechanism genes”. Based on their molecular functions we further classified these “mechanism genes” as either disease causing (leading to neuronal loss/death) or disease alleviating (helps in neuronal survival) agents. Once again, Google and NCBI's PubMed databases were used to identify such previous implications. For easy understanding, the loss versus survival classification is represented in the figures of next sections by highlighting the “mechanism genes” in *purple* or *yellow*, respectively. Using the “mechanism genes” *direct* interaction network was constructed as well as investigated for *integrated* disease mechanism. As will be shown in Section 3 we outlined three possible mechanisms for initiating the Parkinson's disease from extracellular signaling.

## 3. Results

### 3.1 Parkinson's disease direct interaction network

**Figure 4:**
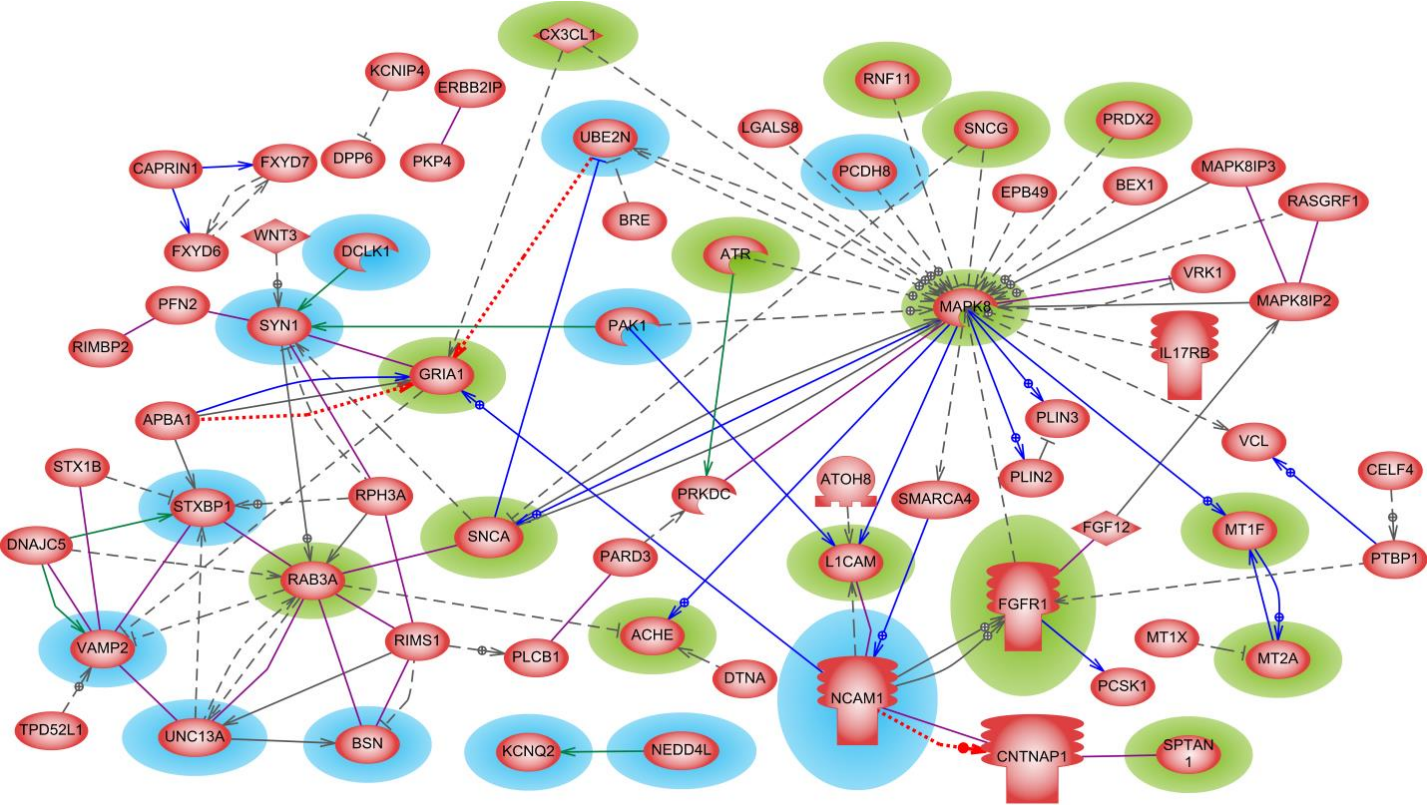
**Parkinson's disease direct interaction network**. The 15 genes/proteins implicated previously in PD pathology are highlighted in green and the 12 genes/proteins of potential interest for that disease are highlighted in blue. Different interactions are represented as follows: regulation – dashed grey, molecular transport – dotted red, co-expression – solid blue, protein modification – solid green, and protein-protein binding – solid purple.

We initiated our Parkinson's disease network analysis using the 267 “seed genes”, selected as explained in Methods and Data. Out of the 267 significantly differentially expressed genes (SDEGs) 67 genes were directly connected to each other by interactions such as regulations, promoter binding, direct regulation, protein modification and miRNA regulation. This interaction network (Figure 4) has a relatively low average node degree of 2.84. Genes like *MAPK8*, *RAB3A*, *STXBP1*, *SYN1* and *VAMP2* are the top five most highly connected nodes with node degree ≥ 7. One of the well-known Parkinson's gene *SNCA* (α-synuclein) was among the top five most influential (betweeness centrality) and highest accessible (closeness centrality) nodes in the network. 15 of the 67 genes/proteins (*ACHE*, *ATR*, *CX3CL1*, *FGFR1*, *GRIA1*, *L1CAM*, *MAPK8*, *MT1F*, *MT2A*, *PRDX2*, *RAB3A*, *RNF11*, *SNCA*, *SNCG* and *SPTAN1*), have already been implicated in Parkinson's disease paradigm either as neuroprotective and therapeutic agents or as disease aggravating ones. In Figure 4, these previously PD-known genes are highlighted in *green*.

Based on their characteristic physiological roles 12 genes (*BSN*, *DCLK1*, *KCNQ2*, *NCAM1*, *NEDD4L*, *PAK1*, *PCDH8*, *STXBP1*, *SYN1*, *UBE2N*, *UNC13A* and *VAMP2*) colored in *blue* in Figure 4 were classified as potentially involved in Parkinson's disease. The molecular functions of some of these candidate genes are summarized here. *NCAM*1 (neural cell adhesion molecule 1) is important in cognitive processes such as learning and memory. It plays a major role in brain immune surveillance system [47]. *NCAM1* also facilitates the release, repositioning, and/or expansion of the synaptic complex. *BSN* (bassoon presynaptic cytomatrix protein), is a scaffolding protein involved in organizing the presynaptic cytoskeleton, the specialized sites where neurotransmitters are released from the synaptic vesicles. (Retrieved on 25-Feb-2013 from http://www.ncbi.nlm.nih.gov/gene/8927). Campbell et al., (2012) [48] have shown that *STXBP*1 (syntaxin binding protein 1) is a vital part of the process of calcium ion–dependent exocytosis in neurons, as well as in neuroendocrine cells. It facilitates membrane fusion and neurotransmitter release. *SYN*1 (synapsin I) is known to be a key player in synapse formation and plasticity [49]. During an action potential (an important part of the neuron firing process), synapsins are phosphorylated by PKA (cAMP dependent protein kinase), releasing the synaptic vesicles and allowing them to move to the membrane and release their neurotransmitter. *VAMP*2 (vesicle-associated membrane protein 2), gene is thought to participate in neurotransmitter release at a step between docking and fusion. A recent study has shown that single nucleotide polymorphisms in *UNC*13A (unc-13 homolog A) gene may be associated with sporadic amyotrophic lateral sclerosis (ALS) [50]. It regulates neurotransmitter release at synapses, including at neuromuscular junctions. α-synuclein was shown to promote disruption of ubiquitin proteasome system [51]. *UBE*2*N* (ubiquitin-conjugating enzyme E2N) targets proteins for degradation via the proteasome. In recent years, synaptic vesicle trafficking defects have been increasingly implicated as an important factor in many PD models, either via direct interactions with the synaptic vesicle (SV) cycling machinery or via indirect effects caused by mitochondrial dysfunction [52]. Even though genes *BSN*, *NCAM1*, *STXBP1*, *SYN1*, *VAMP2* and *UNC13A* are not shown to be directly related to PD, they all seems to play an important role in the regulation as well as the release of neurotransmitters and synaptic vesicles during the SV cycle process.

Additional arguments for considering the above mentioned genes as associated with Parkinson's disease are provided from network perspective. Figure 4 reveals that *BSN*, *STXBP1*, *SYN1*, *VAMP2*, and *UNC13A* directly interact with *RAB3A*, a gene well-known in PD, where *RAB3A* is able to provide substantial rescue against α-syn-induced degeneration of dopaminergic neurons. Besides with *RAB3A*, *SYN1* is also directly connected to *GRIA1* and *SNCA*, two known PD genes. Studies have suggested glutamate receptor (*GRIA1*) antagonists as potential treatment agent for Parkinson's disease [53].

In the *direct* interaction network, potential candidate genes like *PAK1* and *UBE2N* are among the top five nodes with high closeness (visibility) centrality score. Another of the proposed candidate genes *SYN1*, was among the top five hub nodes as well as among the top five nodes with highest betweenness (traffic-influential) centrality score. Being a first-level direct interacting neighbors of a known gene (guilt-by-association), makes also *BSN*, *NCAM1*, *PAK1*, *PCDH8*, *STXBP1*, *SYN1*, *UBE2N*, *UNC13A* or *VAMP2* genes of potential interest in Parkinson's disease. The physiological role these genes play in synaptic vesicle trafficking, neurotransmitter release, and ubiquitination, as well as their other network attributes like being hubs, network traffic-influential and/or monitoring nodes, increases the chance of these genes to be involved in the PD pathology, which reinforces the arguments in favor of their experimental validation.

### 3.2 Parkinson's disease shortest path network (SPNW)

A shortest path network (SP) was built by selecting 105 out of the 267 significant differentially expressed genes (SDEGs), which have a higher chance to be connected to some of the known PD genes (See Methods). Interaction types included promoter binding, protein modification and direct regulation. 193 genes were added by the Pathway Studio 9.0 software to connect the 105 seed genes along the shortest paths between any pair of genes. The connecting genes were examined in sources like OMIM and PubMed, along with Google search to verify whether they have already been implicated or not in PD. In the second case, whether they could be of potential interest in PD diagnosis was decided based on the gene's physiology/molecular characteristics and network location (guilt-by-association).

**Figure 5:**
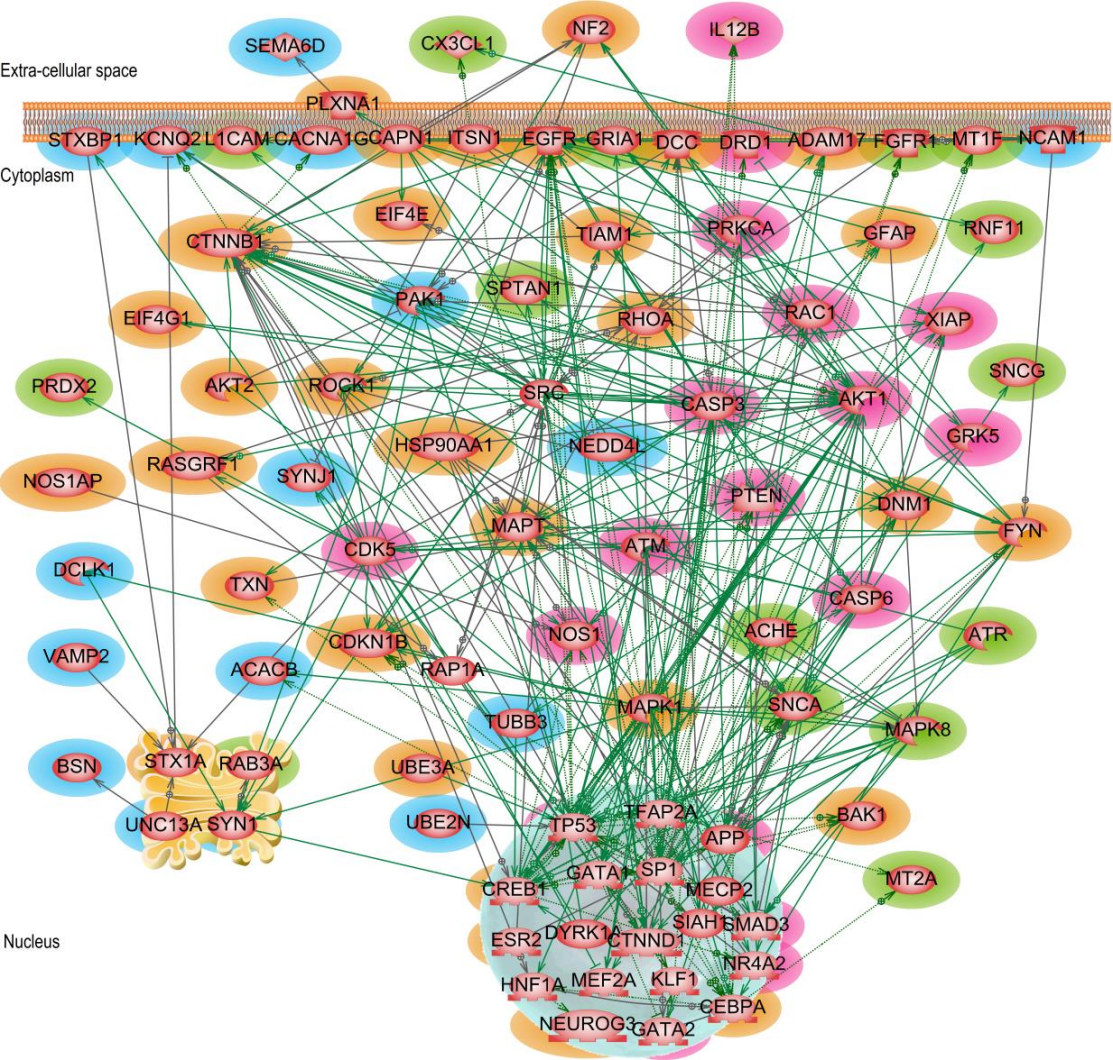
**Parkinson's disease** ***compact*** **shortest path network**. The genes/proteins implicated in PD pathology are highlighted in green and red. The genes/proteins of potential interest are highlighted in blue and orange (green and blue refer to SDEGs, while red and orange to SP network connecting genes, respectively). Different interactions are represented as follows: protein modification – solid green, promoter binding – dotted green and direct regulation – solid grey.

A more *compact* version of this 298 genes SP type network was constructed using only the genes from the four categories of Table 1, and adding few generic genes without which some of the genes of interest would remain unconnected. The *compact* SP network (see Figure 5) is considerably better connected (average node degree 6.79) than the one based on direct interactions. Many of the known PD genes, such as *AKT1*, *CASP3*, *CDK5*, *MAPK1*, *MAPT* and *SNCA* are highly connected in this network. From those, *CDK*5 and *MAPK*1 are among the 10 hub genes (*AKT1*, *CASP3*, *CDK5*, *CREB1*, *CTNNB1*, *EGFR*, *MAPK1*, *SP1*, *SRC* and *TP53*) with node degree > 15. In biomolecular networks highly connected nodes tend to be part of critical functions or pathways, some of the found hubs like *TP53*, *MAPK1*, *AKT1* and *CASP3* being a typical example.

**Table 1:** Summary of genes of interest and genes already known in Parkinson's disease.

Different categories	No. of genes	Node color code in figures
Genes of interest from SDEGs	16	blue
Known PD genes from SDEGs	15	green
Genes of interest in SPNW connecting nodes	36	orange
Known PD genes in SPNW connecting nodes	20	red

The nodes included in the network were then subjected to enrichment analysis using DAVID software tool which systematically maps the given gene list to the associated biological annotation terms. The statistically significant enriched Gene Ontology categories and pathways related to brain and nervous system, assessed with the Benjamini-Hochberg multiple correction, are presented in Table 2. Several clusters of genes were thus identified to be involved in neuron development, differentiation, projection and apoptosis, synaptic transmission, vesicle transport and regulation as biological processes affected by Parkinson's disease. Indeed, many of the enriched genes like *CDK5*, *FGFR1*, *L1CAM*, *NR4A2*, *PRKCA*, *RAB3A*, *RAC1* and *SNCA* have already been studied as mediators, suppressors or regulators of neurodegeneration. Pathways such as ErbB signaling and Neurotrophin signaling are enriched in this PD related gene list. Both these pathways were considered as major avenues to promote survival of dopaminergic neurons [54]. Synaptosomes, axons, and membrane-bounded vesicles are some of the cellular components that are found affected by PD.

**Table 2:** Gene set enrichment analysis of Parkinson's disease compact shortest path network.

Term	Gene Count	Fold Enrichment	Benjamini
GO:0031175∼neuron projection development	16	9.61	1.37E-08
GO:0030182∼neuron differentiation	19	6.67	2.88E-08
GO:0048666∼neuron development	17	7.71	4.11E-08
GO:0048489∼synaptic vesicle transport	8	37.27	1.11E-07
GO:0043005∼neuron projection	16	7.38	2.10E-07
GO:0007268∼synaptic transmission	15	7.74	3.66E-07
GO:0060627∼regulation of vesicle-mediated transport	10	16.01	5.66E-07
GO:0048812∼neuron projection morphogenesis	13	9.38	5.81E-07
GO:0048488∼synaptic vesicle endocytosis	6	65.88	9.59E-07
GO:0007409∼axonogenesis	12	9.56	1.88E-06
GO:0019717∼synaptosome	9	16.71	2.17E-06
GO:0030424∼axon	11	10.92	2.52E-06
GO:0045202∼synapse	14	6.22	7.42E-06
GO:0050804∼regulation of synaptic transmission	10	11.3	7.83E-06
GO:0007611∼learning or memory	9	12.46	1.62E-05
GO:0051588∼regulation of neurotransmitter transport	6	35.48	1.64E-05
GO:0016192∼vesicle-mediated transport	17	4.54	1.83E-05
GO:0046928∼regulation of neurotransmitter secretion	5	36.6	1.80E-04
GO:0001764∼neuron migration	6	14.41	9.20E-04
GO:0021955∼central nervous system neuron axonogenesis	4	40.99	0.002
GO:0031982∼vesicle	14	3.3	0.004
GO:0030665∼clathrin coated vesicle membrane	5	14.89	0.004
GO:0007411∼axon guidance	6	8.62	0.007
GO:0030136∼clathrin-coated vesicle	6	7.17	0.014
GO:0001963∼synaptic transmission, dopaminergic	3	51.24	0.014
GO:0016358∼dendrite development	4	17.57	0.014
GO:0021952∼central nervous system projection neuron axonogenesis	3	38.43	0.023
GO:0012506∼vesicle membrane	6	6.27	0.023
GO:0030425∼dendrite	6	5.81	0.028
GO:0030426∼growth cone	4	11.69	0.035

Table 3 lists the genes identified in our study as possibly related to Parkinson's disease, based on their moderate-to-considerably high connectivity to known PD genes. *CTNNB1* (catenin, beta 1) has the record environment of ten (!) nearest neighbors in the compacted shortest path network (CSPNW, Figure 5) all of which known to be involved in Parkinson's disease (*AKT1*, *CASP3*, *CASP6*, *CDK5*, *CREB1*, *MAPK8*, *NR4A2*, *PTEN*, *RAC1* and *SMAD3*). This makes *CTNNB1* number one candidate gene of interest. This gene, along with Wnt1 and Fzd-1 critically contributes to the survival and protection of adult midbrain DA neurons [55]. In addition, it has a high betweeness centrality which increases its global influence in the network.

**Table 3:** Genes of interest for Parkinson's disease identified by “guilt-by-association” with the known PD-related genes.

Genes of Interest	Interacts with no. of known PD genes
CTNNB1	10
EGFR	6
PAK1	5
CEBPA, CTNND1, ADAM17	4
CDKN1B, KLF1, ROCK1, SYN1	3
AKT2, BAK1, DNM1, DYRK1A, NF2, TUBB3	2
EIF4E, ITSN1, MECP2, NCAM1, NEDD4L, NOS1AP, RASGRF1, RHOA, STX1A, STXBP1, SYNJ1, UBE2N	1

The next strongest candidate for implication with Parkinson's disease is *EGFR* (epidermal growth factor receptor) gene having six PD-related neighbors (*CASP3*, *CDK5*, *PRKCA*, *RNF11* and *TP53*). It is one of the top ten nodes with highest node degree, closeness as well as betweeness centrality scores. This greatly contributes *EGFR* to be one of the critical positions in the *compact* shortest path network with greater visibility and traffic-control. Many studies have shown that *EFGR* signaling play a major role in neurogenesis, neuron survival and maintenance [56–59]. In a recent study, *EGFR* has been suggested as a preferred target for treating amyloid-beta induced memory loss in Alzheimer's disease [60].

Third interesting PD candidate is *PAK*1 (p21 protein (Cdc42/Rac)-activated kinase 1) gene having five PD-related neighbors (*AKT1*, *CASP3*, *CDK5*, *RAC1*, and *TP53*). PAK1 regulates neuronal polarity, morphology, migration and synaptic function [61]. The gallery of Parkinson's disease potentially related genes from Table 3 includes also *CEBPA* (CCAAT/enhancer binding protein (C/EBP), alpha), which interacts with four known PD genes (*GATA2*, *IL12B*, *MT2A*, and *TP53*). *CEBPA* has been shown to bind to the promoter and modulate the expression of leptin, a hormone having easy accessibility to the brain. It is important to note that leptin receptors are expressed in neurons and other brain regions and are known to regulate neural development. Thus, leptin could be a potential drug candidate for neurodegeneration [62].

The *compact* shortest path network included many noteworthy connecting proteins like *APP*, *CREB1*, *HSP90AA1*, *MAPT* and *PTEN* which were previously implicated to play critical roles in many neurodegeneration disease pathogenesis and couple of them were indicated to have neuroprotective mechanism. *APP* (amyloid beta (A4) precursor protein), is the major component of the filamentous inclusions found in the Lewy bodies and Lewy neuritis, the characteristic hallmark features of many neurodegenerative diseases including Parkinson's, Alzheimer's, dementia with Lewy bodies and multiple system atrophy (MSA). Neurodegenerative diseases caused by abnormal aggregations of alpha-synuclein proteins are specially classified as alpha-synucleinopathies [63–66]. Similarly, tauopathies are a class of neurodegenerative diseases that are associated with the aberrant accumulations of tau proteins (*MAPT*) in the brain. Hyper phosphorylated tau proteins are the main component of neurofibrillary tangles (NFTs), another typical pathological feature of neurodegeneration. Tau proteins deformation are found in both genetic and sporadic forms of Parkinson's and Alzheimer's diseases in addition to other neurodegenerative diseases such as progressive supranuclear palsy (PSP), Down's syndrome, Pick's disease [67–70]. Many molecular evidence suggests potential interaction between alpha-syncluein and tau proteins [71]. *PTEN* gene mutations also contribute to the NFT formations and the deregulation of tau phosphorylation [72–74]. Detailed biochemical and genetic studies about *APP*, *MAPT* and *PTEN* molecular processing will be crucial to the development of therapeutic targets to treat many neurodegenerative diseases.

*CREB1* and HSPs were suggested for such therapeutic measures in neurodegenerative disorders. In a mice model study [75], it is indicated that postnatal disruption of *CREB1* along with *CREM* showed progressive neurodegeneration in the hippocampus and in the dorsolateral striatum. This evidences that both *CREB1* and *CREM* can promote nerve cell survival globally in developing brain while more selectively in adult brain. Earlier studies have demonstrated that increase in the expression of HSPs, and especially *HSP70*, by gene transfer or HSPs inducers can reduce the aberrant protein misfolding and inhibit the pro-apoptotic pathway to attenuate dopaminergic neuron degeneration [76].

Besides being major contributors of neurodegeneration process, *APP*, *CREB1*, *HSP90AA1*, *MAPT* and *PTEN* have varying degree of interactions with many known Parkinson's disease genes (see CSPNW, Figure 5). Among these five genes, *CREB1* appears to have major network advantage as being one of the top ten nodes with highest local connectivity, visibility and traffic-influential node in the *compact* shortest-path network. In addition, genes like *APP*, *MAPT* and *HSP90AA1* are among the top 25 nodes with highest connectivity and higher accessibility to all other nodes as measured from their node degree and closeness centrality score. Other genes from Table 3 might also be investigated for possible relations to Parkinson's disease, including the generic genes *MAPK1* and *EGFR*, which are also interacting with many known PD genes.

### 3.4 Integrated Parkinson's disease mechanism

The genes used to construct the *compact* shortest-path network were subjected to Ingenuity's IPA and DAVID *pathway enrichment analysis*, the latter software utilizing KEGG pathway classifications (Kyoto Encyclopedia of Genes and Genomes, http://www.genome.jp/kegg/) [46]. IPA produced 25 enriched pathways vs. 34 for DAVID, and after elimination of the cancer- and infection disease related pathways, the ratio reduced to 18:21. After reviewing Parkinson's disease literature we selected sixteen of the David enriched pathways (Table 4) belonging to categories of signal transduction, cell motility, cell communication, immune system, nervous system and neurodegenerative diseases.

**Table 4:** Enriched KEGG pathways in Parkinson's disease resulted from DAVID analysis.

Term	Gene Count	Fold Enrichment	Benjamini	Genes
hsa04510:Focal adhesion	17	6.62	1.49E-07	PRKCA, EGFR, ROCK1, XIAP, PTEN, SRC, CTNNB1, AKT1, MAPK1, FYN, RASGRF1, RAC1, RHOA, RAP1A, MAPK8, PAK1, AKT2
hsa04520:Adherens junction	10	10.16	6.08E-06	EGFR, FGFR1, MAPK1, FYN, RAC1, RHOA, SMAD3, CTNND1, SRC, CTNNB1
hsa04010:MAPK signaling pathway	15	4.39	4.57E-05	PRKCA, EGFR, FGFR1, TP53, AKT1, MAPK1, CASP3, RASGRF1, MAPT, RAC1, CACNA1G, RAP1A, MAPK8, PAK1, AKT2
hsa04360:Axon guidance	11	6.67	5.10E-05	DCC, MAPK1, ROCK1, PLXNA1, SEMA6D, FYN, RAC1, RHOA, L1CAM, PAK1, CDK5
hsa04012:ErbB signaling pathway	9	8.09	1.12E-04	PRKCA, EGFR, AKT1, MAPK1, CDKN1B, MAPK8, PAK1, SRC, AKT2
hsa04062:Chemokine signaling pathway	11	4.6	6.61E-04	AKT1, MAPK1, ROCK1, TIAM1, RAC1, RHOA, RAP1A, CX3CL1, GRK5, PAK1, AKT2
hsa04310:Wnt signaling pathway	9	4.66	0.002	PRKCA, ROCK1, RAC1, RHOA, TP53, SMAD3, SIAH1, MAPK8, CTNNB1
hsa04722:Neurotrophin signaling pathway	8	5.05	0.004	AKT1, MAPK1, RAC1, RHOA, TP53, RAP1A, MAPK8, AKT2
hsa05010:Alzheimer's disease	9	4.32	0.004	MAPK1, APP, CASP3, NOS1, MAPT, SNCA, ADAM17, CDK5, CAPN1
hsa04530:Tight junction	8	4.67	0.005	PRKCA, AKT1, RHOA, PTEN, SRC, SPTAN1, CTNNB1, AKT2
hsa04115:p53 signaling pathway	6	6.9	0.005	CASP3, TP53, SIAH1, ATR, PTEN, ATM
hsa04370:VEGF signaling pathway	6	6.26	0.008	PRKCA, AKT1, MAPK1, RAC1, SRC, AKT2
hsa05014:Amyotrophic lateral sclerosis (ALS)	5	7.38	0.012	CASP3, NOS1, GRIA1, RAC1, TP53
hsa04540:Gap junction	6	5.27	0.014	PRKCA, EGFR, MAPK1, DRD1, SRC, TUBB3
hsa04620:Toll-like receptor signaling pathway	6	4.65	0.022	AKT1, MAPK1, RAC1, MAPK8, IL12B, AKT2
hsa04810:Regulation of actin cytoskeleton	8	2.91	0.044	EGFR, FGFR1, MAPK1, ROCK1, TIAM1, RAC1, RHOA, PAK1

**Figure 6:**
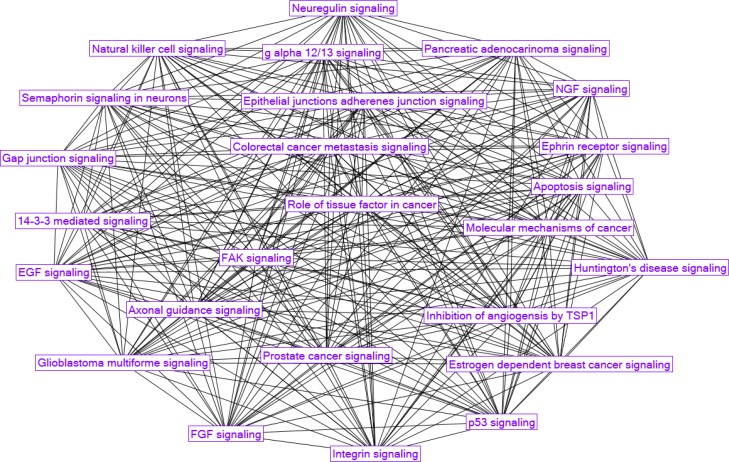
Parkinson's disease enriched canonical pathways as produced by IPA analysis.

Directly shared between IPA and DAVID were the pathways for p53, axonal guidance, gap junction and adherence junction signaling. Many signaling pathways (see Figure 6) including 14-3-3 mediated, neuregulin, semaphorin, ephrin, gap-junction, axonal guidance, as well as different growth factor signaling like EGF/EGFR, FGF, and NGF, were found enriched in Parkinson's disease pathology. This finding extends over the recent report [77]. Neuregulins along with epidermal growth factors play a diverse role in neuronal development and differentiation. Systemic administration of neuregulin-1β1 protects dopaminergic neurons in a mouse model of Parkinson's disease [78]. Semaphorins and ephrins are prominent families of axon guidance cues during normal nerve growth and also after injury. Binding interactions were reported between 14-3-3 proteins Synuclein-alpha and *LRRK2* (leucine-rich repeat protein kinase 2), genes linked to sporadic and familial form of PD [79]. It was symptomatic to find out major neurodegenerative conditions like Alzheimer's disease, Amyotrophic lateral sclerosis (ALS) and Huntington's disease signaling, to be enriched in Parkinson's disease conditions as well. Discovering these overlapping pathways will help to better understand the complex neurodegenerative diseases mechanism and to search for therapeutic agents common for the entire family of these diseases.

**Figure 7:**
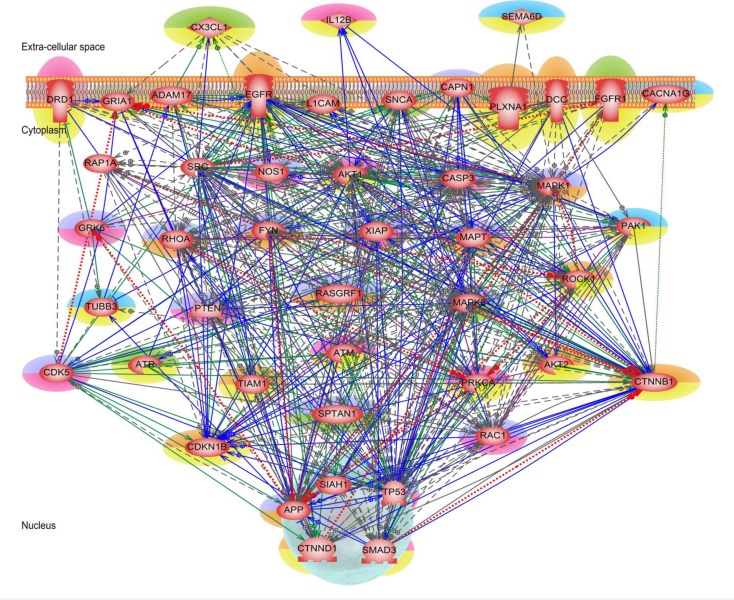
**Integrated Parkinson's disease mechanism**. The 46 genes/proteins found in common in all 16 enriched KEGG pathways. Genes/proteins implicated in PD pathology are highlighted in green/red and those of potential interest are highlighted in blue/orange, where blue and green colored genes belong to the set of significantly modulated genes, while those colored in red and orange are from the set of connecting proteins in shortest path network. Different interactions: regulation – dashed grey, molecular transport – dotted red, co-expression – solid blue, protein modification – solid green, protein-protein binding – solid purple, promoter binding – dotted green and direct regulation – solid grey.

The analysis of genes involved in the selected DAVID/IPA pathways revealed more genes related to Parkinson's disease manifestation, such as *FYN* (protein-tyrosine kinase oncogene belonging to focal adhesion pathway) and *VEGF* (from VEGF signaling pathway). FYN-mediated signaling [80], activates phosphorylation of alpha-synuclein, and the accumulation of this phosphorylated protein in the brainstems of patients with Parkinson's disease is a signature mark of this disease. *VEGF* (vascular endothelial growth factor) is known to promote microglial proliferation, neurogenesis and angiogenesis providing thus neuroprotective effects via both direct and indirect mechanisms with other players of VEGF signaling pathway [81]. This was one more argument to use the 46 genes/proteins found in common in all the 16 KEGG pathways from Table 4 as an essential part of the integrated Parkinson's disease mechanism. The network built on this basis is shown in Figure 7.

The genes in Figure 7 are classified into four categories as being already implicated in Parkinson's disease, such of potential interest to PD, as well as being disease causing (leading to neuronal loss/death) or disease alleviating (helping in neuronal survival. Due to the high network interconnectedness no separation between the loss and survival genes could be detected; the genes appear as part of a single integrated system. Visual inspection of the pathways in KEGG database also revealed that there is no definite compartmentalization of processes within a biological cell. One process/pathway feed into another or multiple pathways, e.g., WnT signaling pathway includes players from MAPK, focal adhesion, adherens junction, and Alzheimer's disease pathways.

In examining the integrated mechanism network three routes emerged for triggering the Parkinson's disease mechanism via one of the extra-cellular ligands *CX3CL1*, *IL12B* and *SEMA6D*. In the first route, *CX3CL1* (fractalkine) together with *DRD1* (dopamine receptor D1) suppresses the expression of ionotropic glutamate receptor *GRIA1*. There is also interaction between *CX3CL1*, *ADAM17* (metallopeptidase domain 17), and *LCAM1* which then follows a downstream path into cytoplasm and to the nucleus for subsequent regulation of gene expression. *ADAM17* and *TP53* activate the expression of the upstream positioned *CX3CL1*. The suppression of microglial activation by fractalkine contributes to neuronal survival. *ADAM17* mediated fractalkine cleavage would ultimately limit activation of microglia and support neuronal survival [82]. There is a two-ways gene expression modulation between *CX3CL1* and *SRC*. Inside the cytoplasm *AKT1*, *CASP3*, *MAPK1*, *MAPK8* genes/proteins are direct downstream targets of *CX3CL1*. Except *GRIA1* and *CASP3*, all other downstream target genes of *CX3CL1* are positively activated by it. Some of the players in the outlined route like *ADAM17*, *CX3CL1*, *DRD1*, *GRIA1*, and *LCAM1* have been claimed in animal model studies as therapeutic targets for Parkinson's disease [19, 21, 83, 84].

Second route is initiated via *SEMA6D* and its receptor *PLXNA1* (plexin A1) which in turn regulates *RHOA* and *AKT1* gene expression inside the cytoplasm. The downstream activity of *MAPK1* in the cytoplasm is also negatively modulated by *SEMA6D*. *SEMA6D*, on its turn can be negatively modulated as upstream target of *PLXNA1*. Apart from *SEMA6D*, *CAPN1* (calpain 1, (mu/I) large subunit) negatively regulates the expression of both *PLXNA1* and *SNCA* and can thus modulate their downstream actions inside the cytoplasm. Semaphorins, secreted proteins involved in the guidance of neuronal and nonneuronal cells, interact with receptor complexes formed by plexins and neuropilins. There is a literature evidence for semaphorins and their receptors to promote or guide neuronal axon projection as therapeutic approaches for treatment of Parkinson's disease [85, 86]. Studies in rodent and cell culture models of PD suggest that treatment with calpain inhibitors can prevent neuronal death and restore functions thus suggesting that calpain inhibition could be a therapeutic strategy in PD [87].

Third route of the proposed integrated Parkisnon's disease mechanism takes place via another extra-cellular ligand *IL12B* (interleukin 12B) which lies upstream to MAP kinases, *RAC1* and *AKT1*, and all these genes negatively regulate the gene expression of *IL12B*. Many studies have suggested that neuroinflammation and activated microglia contribute to neurodegenerative processes. Interleukins alleviate these harmful effects and help in differentiation and survival of neuronal cells that were stressed out by activated microglial actions [88, 89].

Thus, from the integrated disease mechanism network we present a preliminary outline of three possible routes to enhance the survival of the dopaminergic neurons, which could be a source for potential therapeutic targets in Parkinson's disease. A more detailed study will be needed to elucidate this very complex overall mechanism.

### 3.5 Parkinson's disease microRNA regulatory network

**Figure 8:**
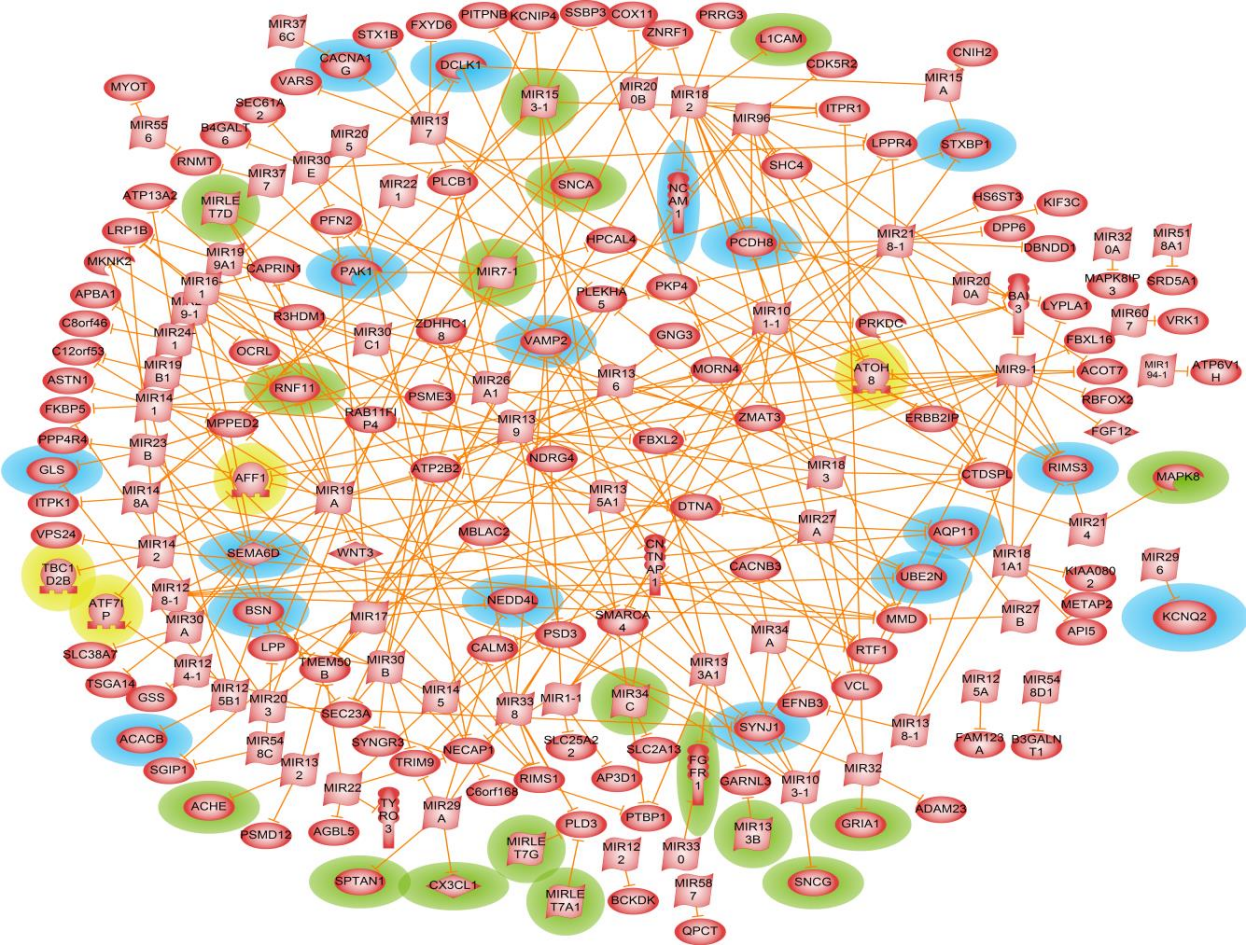
**Parkinson's disease regulatory network**. The genes and miRNAs implicated in PD pathology are highlighted in green and the genes of potential interest are highlighted in blue. Genes that code for transcription factors (TFs) are highlighted in yellow. MicroRNA-mRNA target interactions are represented using solid orange lines.

A *shortest path* network (SPNW) was constructed using all the 267 seed genes and accounting only for their direct microRNA-mRNA target interactions as given in the ResNet 9.0 database of Pathway Studio software. 71 regulatory miRNAs were thus identified (Figure 8).

Table 5 shows the genes of interest in the MicroRNA Regulatory Network (MRN) and how many miRNAs are targeting each gene's mRNA. miR-218-1 was found to be the top player regulating the expression of 16 genes of which three (*PCDH8*, *RIMS3* and *STXBP1*) are of potential interest to Parkinson's disease. In animal model study, it was shown that miR-218-1 is expressed in hippocampus [90], where volumetric MRI imaging study have found a progressive volume loss in PD human subjects [91]. Other microRNAs like miR-29a, miR-132, miR-133a1, miR-182, and miR-330 were found to regulate the expression of the known Parkinson's related genes *ACHE*, *CX3CL1*, *FGFR1*, *L1CAM*, and *SPTAN1*. Being direct interacting partners with known PD-related genes some of these miRNAs could be considered as potential regulatory targets in Parkinson's disease mechanism.

**Table 5:** Genes of interest determined from Parkinson's disease microRNA regulatory network.

Genes of Interest	Targeted by No. of miRNAs
RIMS3, SEMA6D, SYNJ1	7
PCDH8	6
AQP11, VAMP2	5
DCLK1, PAK1	4
BSN, NCAMP1, STXBP1, UBE2N	3
CACNA1G, GLS, NEDD4L	2
ACACB, KCNQ2	1

The microRNA regulatory network also incorporates some of the already implicated miRNA's (miR-133b, miR-153, miR-34c, miR-7 and miR-let-7) mediated translation regulations of *DJ-1*, *PARKIN*, *PITX3* and *SNCA*. These genes are relevant to PD pathophysiology being shown to regulate dopaminergic neuron differentiation and activity, oxidative stress mediated cell death, and mitochondrial energy production dynamics [77–79].

On further examination, microRNA regulatory network revealed that the expression of candidate genes like *RIMS3*, *SEMA6D* and *SYNJ1* was tightly regulated by multiple miRNAs. *RIMS3* (regulating synaptic membrane exocytosis 3) and other RIM family members are generally believed to be RAB3 isoforms (RAB3A/B/C/D)-specific effectors that regulate synaptic vesicle exocytosis in neurons and in some endocrine cells [95]. Release and re-uptake of neurotransmitters in the synaptic junction is a highly coordinated process and *RIMS3*, and *RAB3A* along with other proteins play an important role during neurotransmitter release.

The gene expression of the extra-cellular ligand *SEMA6D*, proposed as one of three initiators of the integrated Parkinson's disease mechanism (Figure 7), was found in our miRNA regulatory network to be regulated by seven miRNAs (miR-124-1, miR-128-1, miR-16-1, miR-19a, miR-23b, miR-30a and miR-9). Some of those like miR-124-1, miR-128-1 and miR-9 have been previously shown of importance for Alzheimer's disease neuropathology being abundantly expressed in Alzheimer hippocampus [96]. This may be considered as one more sign for the possible existence of common regulatory mechanisms in neurodegenerative diseases.

Another highly microRNA-regulated gene is *SYNJ1* (synaptojanin 1) (see Table 5), a polyphosphoinositide phosphatase found enriched in the brain and located at nerve terminals, as well as associated with synaptic vesicles and coated endocytic intermediates. Synaptojanins were suggested to accelerate the synaptic vesicle recovery/trafficking process at the synapse [97]. Dysfunction of synaptic transmission and membrane trafficking are implicated in PD. Based on its molecular function, *SYNJ1* could play a role in Parkinson's disease molecular mechanism.

Finally, in addition to miRNA mediated regulation, the network also included four genes (*AFF1*, *ATF7IP*, *ATOH8* and *TBC1D2B*) that encode for transcription factors (TFs). These significantly differentially expressed TFs indicate a possible integrated TF/miRNA regulation of the transcription of Parkinson's related genes.

## 4. Summary

The microarray expression data used in our study were a combination of data produced and interpreted by different authors [33, 34] and referring to different regions of brain. With a long-term aim to search for a common molecular mechanism for neurodegenerative diseases, we renormalized the data for a better comparability. Then, a number of specific biomolecular networks were built and analyzed in a variety of ways. As a result, while confirming some of the previous finding, including part of the novel predicted Parkinson's genes, more such PD-related genes were proposed in this work based on guilt-by-association analysis and accounting for the importance of certain nodes in network topology.

As well known, the guilt-by-association approach is based on analysis of the nearest network neighborhood of genes with proved function in the search of interest. Many Parkinson's disease genes were listed in the OMIM database. However, our list of SDEGs in *all* three Parkinson's disease datasets used in this study did not include all of the OMIM PD-related genes, missing such genes like *LRRK2*, *PARK2*, *PARK7*, *PLA2G6*, *PINK1* and *UCHL1*, while *PINK1* and *UCHL1* were still both significantly expressed in medial Substantia nigra, and *UCHL1* also in lateral Substantia nigra, but not in all three brain tissue types. We found that the log fold-change of *PARK2*, *PARK7* and *PLA2G6* was only around 0.03, which was not significant enough to detect changes in gene expression. Affymetrix HG-U133A GeneChip did not contain probe for *LRRK2* gene but instead included *LRRK1* gene probe. Again, *LRRK1* did not meet the criteria for “seed genes” list since it did not show strong differential gene expression and its log fold-change was also only around 0.03. While the lack of statistically significant presence of the above mentioned PD-related genes could possibly be attributed to the loss of expression intensity in the post-mortem brain samples compared to a functioning brain, in this study we focused our attention mainly to the genes showing considerable change in all three selected Parkinson's disease brain tissue samples.

Despite the reduced base of 15 known PD-genes needed for the guilt-by-association predictions we were able to identify from our *direct interaction network* *SYN1* neighboring three known PD genes, followed by *UBE2N* and *NCAM1* with two and *BSN*, *PAK1*, *PCHD8*, *STXBP1*, *UNC13A* and *VAMP2* with one such neighbor as novel Parkinson's disease candidate genes. Second-level interacting partners generally have much lesser chance to be included in the list of candidate genes. However, this chance may increase for some genes known to show certain functions that may be related to the disease of interest. Such is the case with *DCLK1* gene via its role in synaptic plasticity and neurodevelopment and as being first neighbor of *SYN1*. Another group of novel PD gene candidates was found from similar analysis of the *shortest path* network. Such is the case with *NEDD4L*, *SYNJ1*, *TUBB3* as direct partners, and *ACACB*, *CACNA1G*, *KCNQ2*, and *SEMA6D* as second-level partners to already known PD genes. All 17 genes listed here are significantly differentially expressed in PD.

Our network analysis indicated that apart from the strongly differentially expressed genes some connecting genes/proteins from the shortest path networks could be of similar importance in the deregulation of the disease mechanisms. Considering such *connecting* genes/proteins via their guilt by associations to already known PD genes we concluded that *CTNNB1*, *EGFR*, *ADAM17*, *CEBPA*, *CTNND1*, *CDKN1B*, *KLF1*, *ROCK1* and *TIAM1* could also be genes of potential interest in Parkinson's disease realm. Some of the genes of this list were found to play an important role in network topology. Thus, *CTNNB1* and *EGFR* are among the top ten highly connected nodes (with degree > 15), among the top ten nodes with higher accessibility to all other nodes as assessed by the closeness centrality, and among the top ten traffic influential nodes in the network as judged by their betweenness centrality. Genes like *ADAM17*, *CEBPA* and *CTNND1* are among the top 25 high connectivity nodes (with degree ≥ 8) and also among the top 25 traffic-influential nodes in the network. Besides helping in identifying novel PD-related genes, the same line of network analysis has shown that *APP*, *MAPT* and *PTEN*, well-known contributors of many other neurodegenerative diseases including Alzheimer's, MSA, Pick's, PSPs etc., are important *connecting* genes/proteins in the Parkinson's shortest-path network. Finding such genes with common role in neurodegeneration process reinforces our study goal.

We have also added another seven to the numerous miRNAs already known to affect the expression of PD-relevant genes [92–94]. With caution, because some of their regulatory interactions are not yet validated, we predict that miR-132, miR-133a1, miR-181-1, miR-182, miR-218-1, miR-29a, and miR-330 could be of interest as potential regulators in Parkinson's disease mechanisms, due to their direct interaction with known PD related genes. Further investigation of the above mentioned miRNA-related regulatory interactions of candidate and known PD-genes would deepen our understanding of the molecular mechanisms of complex diseases like Parkinson's. Examining the microRNA regulatory network, one may conclude that disease pathogenesis is complex enough and requires regulatory mechanisms mediated via both protein-coding genes and the small noncoding microRNAs.

All genes listed in this summary were shown through gene set enrichment analysis to be key players in various cellular pathways and mechanisms like neuron development and differentiation, synaptic transmission, vesicle transport and endocytosis, apoptosis, and memory/learning, which are altered in the underlying Parkinson's pathophysiology and the potential compensatory responses. Moreover, enrichment of Alzheimer's, ALS and Huntington's disease signaling pathway was found to take place in PD brains as well. This supports the views for the presence of an underlying common mechanism for all neurodegenerative diseases.

In the final stage of our systems biology approach to Parkinson's disease we used the KEGG pathways found enriched by DAVID analysis along with the enriched canonical pathways from IPA analysis to build an integrated mechanistic Parkinson's disease network containing 46 genes. Three routes of triggering PD molecular mechanisms were identified on this basis proceeding from signaling initiated via the extra-cellular ligands *CX3CL1*, *SEMA6D* and *IL12B*. Further analysis of these routes could reveal novel therapeutic targets for Parkinson's disease. Yet, the above findings could be considered only as the tip of the iceberg in understanding the intertwined nature of the highly complex neurodegenerative diseases.

## Supplementary Material

A Network View on Parkinson's Disease
